# Lifelong Learning With Type 1 Diabetes: The Lived Experiences of Adults Diagnosed in Younger Years

**DOI:** 10.1177/26350106251361371

**Published:** 2025-08-05

**Authors:** Charlotte Gillrell, Peter Hellman, Malin Axelsson, Anne Wennick

**Affiliations:** Lindeborg Primary Care Centre, Region Skåne, Malmö, Sweden; Faculty of Health and Society, Department of Care Science, Malmö University, Malmö, Sweden; Faculty of Health and Society, Department of Care Science, Malmö University, Malmö, Sweden; Faculty of Health and Society, Department of Care Science, Malmö University, Malmö, Sweden

## Abstract

**Purpose:**

The purpose of this study was to illuminate the lived experiences of lifelong learning in self-care from the perspective of adults diagnosed with type 1 diabetes in younger years.

**Methods:**

In 2020 to 2021 a hermeneutic phenomenological study was conducted in Sweden based on individual conversational interviews. Participants in the study were 20 consecutively chosen adults diagnosed with type 1 diabetes, ages 25 to 75 years (median 44.5 years) and with an illness duration of 10 to 61 years (median 26 years), recruited purposively via social media.

**Results:**

The analysis of participants’ lived experiences of lifelong learning in self-care generated the overall theme “making meaning of type 1 diabetes as a lifelong illness”; this process was constantly challenged or triggered by all other ongoing or occurring processes in the participants’ everyday lives. More precisely, the participants likened this to having a ticket for a lifelong journey of personal learning, largely informal, characterized by a continuous reconstruction of one’s understanding of the illness and the necessary self-care while having to acknowledge, understand, manage, and endure diabetes.

**Conclusions:**

Study findings suggest that lifelong learning in diabetes self-care in everyday life means learning how to become and remain a lifelong learner in the trajectory of diabetes. Thus, continuously learning how to manage self-care in different situations throughout life helps those diagnosed with a lifelong illness to construct and reconstruct living with the illness into a meaningful life.

Type 1 diabetes (referred to in this article simply as “diabetes”) is described as a life-changing illness that the affected individual has to learn how to live with.^1-3^ The rationale for this description is the individual’s need to learn how to accept the illness as a physical change entailing a new understanding of oneself as a person with diabetes.^4,5^ However, learning to live with diabetes is a complex process comprising both physical and emotional challenges^6^ that are lifelong.^7^ Although previous research describes living with diabetes as living with a lifelong illness,^8,9^ there is only limited research stressing that the learning process too is lifelong.^7^

When assuming a learning approach rather than a teaching approach, the focus alters from the institutional context of educating the person to a focus on the inner development of the individual; thus, the focus is on the person rather than the content.^10^ In addition, individuals diagnosed as having diabetes would, in accordance with this approach, not only learn but also be able to integrate their personal experiences of the illness as part of a lifelong development process. Consequently, to become a lifelong learner about diabetes, the person needs to change focus from striving to obtain knowledge about diabetes to developing the ability to integrate diabetes and diabetes self-care into everyday life. Thus, the goal is not only to learn facts about the illness but also to learn to develop one’s identity in a social context.^11^ Learning from a lifelong perspective also means an altered understanding created through learning over time at different stages of life^12^ and learning widely within every possible situation in life and throughout life.^10^ Thus, based on a lifelong perspective, adulthood is a series of situations and transitions, each of which drives the person into uncharted territory and triggers the motivation to reconstruct one’s identity again.^13^ Applying this to a person with diabetes, a lifelong learning perspective implies that the individual’s understanding of diabetes and self-care may alter over time and at different stages in life.

The concept of self-care can be described as a process of maintaining health through health-promoting practice and managing illness.^14^ It focuses on long-term illness (eg, diabetes), and its core elements are: self-care maintenance, focusing on well-being; self-care monitoring, focusing on body monitoring; and self-care management, focusing on evaluation of changes in bodily and emotional signs.^15^ Self-care monitoring is a distinct concept bridging the gap between self-care maintenance and self-care management. Thus, self-care is essential when living with diabetes because these individuals’ knowledge of self-care and their own abilities and their emotions shapes their experiences when interacting with their surroundings. This personal development occurs when the experiences are processed, learned, and integrated into the person’s being, which means “being” and “becoming” in a lifelong process.^10^ In the case of a person with diabetes, this would imply being and becoming through a continuously reconstructed understanding of diabetes and self-care as an ongoing process in everyday life and throughout life.

Although lifelong learning is well established in educational literature,^10,12,16^ it is traditionally not discussed in the context of diabetes self-care. Furthermore, the way that the understanding of self-care and diabetes alters over time has barely been studied. This became evident when reviewing the existing literature on lifelong learning in diabetes self-care, which resulted in only 1 longitudinal qualitative descriptive study^7^ describing learning in diabetes self-care from an individual point of view; that study found that learning from a recent diagnosis develops over a 3-year period. Because there is limited research on lifelong learning in diabetes self-care for an illness duration exceeding 3 years, the purpose of this study was to illuminate the lived experiences of lifelong learning in diabetes self-care from the perspective of adults diagnosed with type 1 diabetes in younger years.

## Methods

### Study Design

This study was designed as a qualitative inductive study using individual conversational interviews with adults diagnosed in younger years to illuminate their lived experiences of lifelong learning in diabetes self-care. The methodological approach chosen was hermeneutic phenomenology influenced by van Manen,^17^ who outlined 4 existential dimensions belonging to the fundamental structure of the lifeworld: lived body, lived time, lived human relations or other, and lived space. These 4 dimensions allow a deeper understanding of the lived experiences of lifelong learning in diabetes self-care and thus provided the rationale for choosing hermeneutic phenomenology as the methodological approach. This study was approved by the Swedish Ethical Review Authority (Reg. No. 2019-06330).

The study was conducted in Sweden in 2020-2021, when the praxis was that those diagnosed with diabetes should be offered care by health care professionals specially trained in diabetes care. When first diagnosed, a standardized patient education based on the national guidelines for diabetes care is offered,^18^ which are consistent with the 2022 National Standards of Diabetes Self-Management Education and Support.^19^ To achieve the best possible treatment outcome, it is recommended that this patient education is led by health care professionals with both subject knowledge and teaching skills. In addition, the education should be adapted to the cultural background of the individual with diabetes, in terms of diet and view of health and illness, and should also satisfy the individual’s specific needs for more knowledge.

### Participants

Of the 28 adults in a purposive sample from social media sources who responded to one of the study authors (MA) for further information, 20 gave their written informed consent to participate. The inclusion criteria were being 18 years of age or older, diagnosed with type 1 diabetes, and able to read and understand Swedish. To select the participants, a recruitment notice about this study was posted on 2 different group sites on Facebook, both addressing diabetes. Permission to advertise was obtained from each site administrator. Furthermore, an embedded social feed about this study was posted for 2 weeks on Facebook to all users 18 years of age or older. Those consenting to participate were 24 to 75 years of age (median 44.5), had been diagnosed with diabetes at the age of 2.5 to 30 years, and had an illness duration of 10 to 61 years (median 26; Table 1).

**Table 1.** Overview of Participant Demographics

Variables	
No. of participants	20
Age (y), range (median)	24-75 (44.5)
Age at diagnosis (y), range (median)	2.5-30 (11.5)
Diabetes duration (y), range (median)	10-61 (26)
Sex (n)	
Male Female	6 14
Education level attained (n)	
9-y compulsory school Upper secondary school Vocational school College University	1 2 5 1 11
Household status (n)	
Single household Married or cohabiting	5 15
No. of participants with children	5
Occupation (n)	
Employed Student Retired	14 3 3

### Data Collection

Individual conversational interviews were conducted at the participants’ convenience: either by telephone (n = 18) or via Zoom (version 6.2.50939; n = 2). One of the authors (CG; PH; MA or AW) conducted each interview, and with the participant’s consent, the interview was recorded on audio file and transcribed verbatim in close connection with the interview. During the interviews, the participants sat undisturbed in a room of their choice at home or at their workplace. Before the interview started, the participants were asked basic demographic questions (Table 1), and before asking the first interview question, the interviewer read out the information letter and stated that the participant had given written informed consent to participate in the study. The participant was then invited to ask for anything to be clarified. When all such questions (if any) had been answered, the interview started.

The interviews were conducted following an interview guide based on previous research and literature in the field, with the questions calibrated for this study in a pilot interview. The opening question asked participants to narrate in their own words their experiences of what they had learned about diabetes when they were first diagnosed (Table 2). The interview then proceeded to explore these experiences in depth by asking if there were situations they found particularly difficult when first diagnosed with diabetes, how these situations had affected their diabetes self-care over time, and how they had best learned to manage diabetes over time in different situations. Follow-up questions were asked only for the purpose of clarification and to elicit discussion to highlight the phenomenon “lifelong learning in diabetes self-care.” No further guidance was given during the interview, and the participants covered the experiences that they considered relevant in no specified order. The interviews lasted as long as the participants needed, resulting in a total of 223 transcribed pages, ranging from 5 to 16 pages (mean 10.5) per participant.

**Table 2.** An Overview of the Interview Guide

Interview guide questions	
Opening question	Could you tell me what you learned about diabetes when first diagnosed?
Interview topics	Situations found particularly difficult Situations affecting self-care over time Best way to learn to manage diabetes over time in different situations
Probing questions	Can you tell me more about that? Why do you think that is? In what way is that so?

### Data Analysis

The analysis began during the interview, with the interviewer (CG; PH; MA or AW) actively listening and thinking about the meaning of what the participant was saying. The analysis then continued with each interview transcript being anonymized and compared with the recorded audio file for accuracy of the transcription and to note additional features of the interview (eg, voice inflection, laughter, or onset of crying). Subsequently, the first (CG) and last (AW) authors read and reread each transcript independently. Once an overall understanding of the transcripts was obtained, these authors (CG and AW) independently analyzed the interviews using a selective reading approach as described by van Manen.^17^ More specifically, phrases in each interview that seemed essential or revealing about the participants’ lived experiences of lifelong learning in diabetes self-care were underlined, and tentative theme labels that briefly described the content were written in the margin of the transcript. To gain a deeper insight and understanding of the participants’ lived experiences, preliminary themes and thematic descriptions were first discussed collaboratively by these authors (CG and AW) and then among all authors (CG, PH, MA, and AW) until consensus was reached (Table 3). Finally, all the transcripts were reread by the last author (AW) to check if any lived experiences had been missed and to confirm that the analysis reflected the participants’ narratives.

**Table 3.** Extract From the Data Analysis Process

Interview phrases revealing the participants’ lived experiences of lifelong learning in diabetes self-care	Subtheme	Overall theme
“I don’t strive for a straight blood glucose curve, because then I’m not living. But I aim to keep my blood glucose within my target range.” (IP 1)	Managing diabetes	Making meaning of diabetes as a lifelong illness
“Previously, I didn’t dare go out or go to bed. I had to eat extra to dare to sleep. Now I know that the insulin delivery is slowed down if my blood glucose gets too low during the night.” (IP 14)	Managing diabetes	

Abbreviation: IP, interview person.

### Reflexivity

A personal inventory was made by each author to raise their awareness of preconceived opinions and perceptions that could subjectively influence the analysis process. Based on these inventories and subsequent field notes, the researchers engaged in a continuously reflexive dialogue throughout the study, from its conception to the research outputs. Their experiences come from working as a registered nurse (CG, PH, MA, and AW), registered nurse specialized in diabetes care (CG), and registered pediatric nurse (AW). In addition, the authors (PH, MA, and AW) have been doctors of medical science for many years, and the last author (AW) is well versed in hermeneutic phenomenology.

## Results

**Figure 1.**
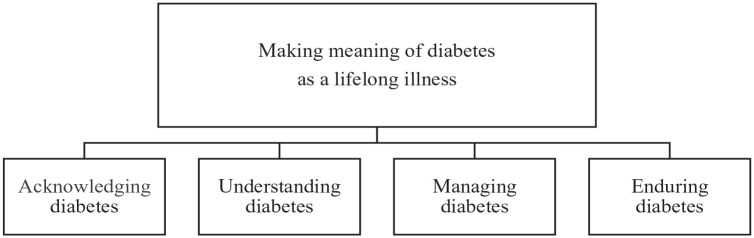
An overview of the overall theme and related subthemes illuminating the lived experiences of lifelong learning in diabetes self-care from the perspective of adults diagnosed in younger years.

The participants’ lived experiences of lifelong learning in self-care were identified in the overall theme as a process of making meaning of diabetes as a lifelong illness. This process was constantly challenged or triggered by all other ongoing or occurring processes in the participants’ everyday experience throughout life. The participants described this as having received a ticket to a lifelong journey of personal learning, largely informal, characterized by a continuous reconstruction of one’s understanding of the illness and diabetes self-care at the same time as their diabetes evolved, healed, and changed their lives. For the journey to begin, the participants had to learn to recognize themselves as a person diagnosed with diabetes, which formed the subtheme “acknowledging diabetes.” Next on this journey was for them to understand how the body with diabetes operates on an everyday basis, which formed the subtheme “understanding diabetes.” Along with this followed the realization that everyday actions affect plasma glucose levels, stressing the importance of the participants’ ability to manage their self-care, which formed the subtheme “managing diabetes.” The outcome of every single transition during this journey, normally occurring throughout life, was influenced by the participants’ ability to endure living with diabetes, which formed the subtheme “enduring diabetes” (Figure 1). To enhance the confirmability of the findings, the subthemes are illustrated in the following with quotations from the original transcripts, marked “IP” (interview person) and a number to show which interview the extract originates from without identifying the individual participant.

### Acknowledging Diabetes

Receiving the diagnosis implied an insight that life would never again be the same as before. Furthermore, at this point, the participants recognized that the acknowledgment of diabetes was highly influenced by personality and was a process that could last for several years. During this process, a positive and solution-focused personality was beneficial because it triggered their feeling of freedom and of being free to make choices regarding diabetes self-care and about other aspects of life. The participants reported that external social support strengthened their ability to visualize a future with diabetes, whereas a lack of social support made it harder to visualize. Provided that the participants accepted the illness and integrated diabetes self-care into their everyday lives, they felt that they had room to maneuver, which was instantly restricted if they had not accepted their illness: “To live my life the way I wanted and not feel alienated because of my diabetes I didn’t bother to control my plasma glucose levels or take insulin when amongst people. Sometimes I forgot to take insulin altogether” (IP 26).

To preserve room to maneuver in their lives, participants sometimes chose to override the illness in various everyday life situations or to opt out or forget all about self-care despite any short- or long-term consequences.

### Understanding Diabetes

Participants described the understanding of self-care in everyday life as a duality. It entailed both having to learn how the illness affected their lives and conversely, how a person’s life affects the self-care management of bodily and emotional changes. To increase their understanding, some preferred to reflect on the illness and its self-care with others diagnosed with diabetes because shared experiences offered valuable insights. However, others commented that self-care could not be taught in interaction with an equal because they considered diabetes to be an individual illness affected by ones’ own everyday life:

There is always something happening in my life that is new and affects life with diabetes. Diabetes is an individual illness at an extreme level . . . [pause] . . . So, for the management of diabetes to work in my everyday life, a continuous reinterpretation of my experience is needed throughout life. (IP 26)

The participants emphasized that the motivation to learn about self-care had to come from themselves. They then learned through trial and error and through their accumulated experiences. Thus, to determine whether actions were needed, they had to have a compound understanding of their individual life situation combined with an understanding of diabetes self-care. Learning to understand which actions one needed to take to improve one’s self-care were facilitated, according to the participants, by abilities such as independent learning, reflection, and analysis. To further facilitate understanding and to put their intentions into practice, they recommended an approach of indomitability and courage to dare to live with diabetes. Lastly, for them, learning about diabetes self-care to improve their well-being was not static but occurred throughout life.

### Managing Diabetes

The participants described how to be able to live with diabetes, they had to undertake management of the illness, which implied having to undergo a learning process throughout life. More explicitly, they had to learn that a plasma glucose within target levels provides control of one’s body and of one’s inner self. Thus, when they achieved control, it positively affected their well-being, mood, and everyday life, which were all inhibited if they lacked complete control:

My life is rock hard. I eat the exact same amount of carbohydrates every meal, exercise the exact same every day. On a few days when I need to break the routine, it punishes me instantly, as my blood glucose levels then swing. (IP 30)

Loss of control during an episode of hypoglycemia could trigger their feelings of anxiety and fear of dying. Although some participants perceived increased knowledge of hypoglycemia as improving their self-control, others felt that it created a loss of self-control. Consequently, the optimal situation was when they achieved an even plasma glucose level because this gave them increased focus on their everyday life as opposed to focusing on the illness, especially because varying plasma glucose level resulted in constantly having to monitor it and adjust their insulin dosage accordingly, which could limit their room to maneuver in everyday life. Managing self-care during fluctuating plasma glucose presumed an ability to respond to varying levels without always being able to detect the cause of the fluctuation. Thus, learning to manage self-care was perceived as a full-time job throughout life.

### Enduring Diabetes

The participants described diabetes as a lifelong challenge they had to endure. Their patience was tested by the constant need to monitor the illness, which was why they put self-care management on hold every now and then to be able to endure life with diabetes. Perhaps the hardest thing they reported about having diabetes was resigning themselves to the realization that they would never master diabetes self-care management fully. At the same time, their ability to tolerate living with the illness was strongly influenced by how they managed transitions throughout life:

In different periods of my life, I have dealt with diabetes differently. When I met my husband 10 years ago, I realized that I could no longer walk around putting on a brave face. My husband and I share everything about my diabetes. (IP 6)

The participants commented that to be able to live with diabetes and to endure the illness, they had to engage in a change process that developed over time. This process required different self-care management strategies at different points in time. The process was ultimately facilitated if the self-care management focus was long term rather than short term, for example, focusing on individual plasma glucose levels: “How I manage my diabetes as a child, adolescent, and adult is different, whereas the change in how I manage my diabetes is happening slowly and gradually. Changes are almost invisible” (IP 24).

The participants had found it harder to endure their diabetes when caregivers or relatives prohibited candies or stressed the risk of future complications because this made them sad and affected their social life. If the participant’s ability to endure diabetes was negatively affected, their well-being (self-care maintenance) was also negatively affected. Whereas some learned how to endure the illness retrospectively, others learned how to do it prospectively: “The choices made today could affect my health in 30 years. I only understand the consistency afterwards. Diabetes is a mentally and physically stressful illness. I have diabetes every day, so sometimes I’m a little tired” (IP 21).

The participants wanted to achieve prospective endurance of the illness and well-being (self-care maintenance) developed through intellectual calculation of risks and benefits, which, as they pointed out, takes a long time to learn.

## Discussion

From the perspective of the 20 adults diagnosed in younger years who were interviewed in this study, lifelong learning in diabetes self-care is part of a learning process of acknowledging, understanding, managing, and enduring diabetes to make meaning of it as a lifelong illness. The following reflections on the findings of this study are based on the 4 existential dimensions outlined by van Manen^17^ as belonging to the fundamental structure of the lifeworld: lived body, lived time, lived human relations or other, and lived space. “Lived body” (corporeality) refers to the fact that we are always bodily present in the world. This dimension was described in the subtheme acknowledging diabetes when the participants commented that the learning process of acknowledging diabetes could last for several years. Previous research has argued that the process of accepting diabetes is complex and is continuously learned through trial and error.^20^ In this study, it was linked to the room to maneuver with the illness in everyday life. When they acknowledged their diabetes, their room to maneuver evolved, and when they failed to accept the illness or consistently integrate diabetes self-care into their everyday lives, they immediately found their room to maneuver limited; this limitation could result in hypoglycemia and a loss of bodily control involving one’s whole existence being challenged and adversely affecting one’s learning process and transitions through life. In contrast, if the individual with diabetes understands the bodily signals, it can be a tool for acknowledging the illness and understanding it.^20^ Thus, learning to live with diabetes refers to taking responsibility for the illness on a short-term and long-term basis and taking responsibility for one’s learning in a way that promotes health and well-being.^21,22^ Because the first step in the learning process to make meaning of diabetes as a lifelong illness focuses on acknowledging diabetes, it may be beneficial if health care professionals could assume a learning approach to assist persons diagnosed to acknowledge their illness in everyday situations and over time. If not, there is a risk that the parties will find themselves on different understanding horizons regarding what it means to live with diabetes, without meeting in thought.

Lived time (temporality) refers to subjective time as opposed to clock time or objective time.^17^ This dimension was described in the subtheme enduring diabetes. Continuously learning to live with a lifelong illness such as diabetes implies conscious efforts to prevent disjuncture from everyday routines at the same time as being compliant to self-care regimens to prevent future illness-related complications. Previous research describes lived time as having an awareness of being different than before and recognizing changes by reflecting on earlier situations.^5^ In addition, it implies an awareness of a changed future with a need to make decisions and take actions related to one’s immediate future and many years ahead.^5^ Thus, the ultimate purpose is not simply to acquire knowledge as an adult learner but to learn to be and become a lifelong learner.^10^ Whereas acknowledging is the first step of the learning process to make meaning of diabetes as a lifelong illness, enduring is the last step. Seeing that learning is integral when managing a lifelong illness, this last step may perhaps be reached if health care professionals assist persons diagnosed to adopt a tailored self-reflection technique (eg, journaling, talking with family members or friends, practicing mindfulness, and spending time alone) to focus on topics matching the person’s learning needs. Such techniques may support persons with diabetes if continuously evaluated and adequately adjusted for the initiative to continue to fulfill their learning purpose throughout the person’s life.

Lived human relations (relationality) refers to the lived relationships we maintain with others in the interpersonal space that we share with them.^17^ This dimension was described in the subtheme managing diabetes. Managing self-care in the sense of bodily and emotional control is essential because fluctuating plasma glucose levels make it harder to live with diabetes and can lower the individual’s well-being. Therefore, when monitoring plasma glucose levels, one must make choices regarding adherence and/or adjustment of self-care management according to one’s life situation, family, friends, and coworkers or the like, which is an ongoing learning process throughout life.^23^ It is also important to receive support from health care professionals on how to monitor the illness, maintain metabolic control, and act on changes, which are vital elements of self-management.^15^ One’s life situation, family, friends, and coworkers or the like are not likely to be static throughout one’s life. Thus, it may be beneficial if health care professionals were to invite persons diagnosed to reflect on experienced everyday situations to discern who is providing support when managing the illness and subsequently, to jointly reflect on how the illness can be communicated to others for increased everyday support.

Lived space (spatiality) refers to the ways we experience spatial dimensions (felt space) of our everyday existence.^17^ This dimension was described in the subtheme understanding diabetes. A good balance in relation to diet, physical activity, insulin, and plasma glucose levels is needed to experience freedom when living with diabetes. However, not only a balanced lifestyle but also socio-environmental factors produce an experience of freedom compared to being illness-controlled.^24^ An interview study aiming to understand the experiences of growing up with diabetes from the perspective of 35 adults showed that the illness does not have to limit one’s activities.^2^ This is not entirely consistent with the findings in our study because our participants pointed out that taking part in some activities implied taking a calculated metabolic risk, which they sometimes found unacceptably high, thus limiting the scope of their activities. Because diabetes is a lifelong illness, it may be beneficial if health care professionals could assume a learning approach to assist persons diagnosed to understand how they can experience freedom when living with diabetes instead of being illness-controlled and only focusing on metabolic control.

### Strengths and Limitations

Various steps were taken throughout this study to enhance its trustworthiness, in accordance with Lincoln and Guba.^25^ First, to ensure credibility and increase confidence in the authenticity of the results, a collaborative analysis was conducted by all 4 authors. In order not to burden the participants any further, none were invited to comment on the interview transcripts or the findings. Instead, the transcripts were cross-checked against the audio files by all 4 authors for accuracy, and after the final analytical consensus had been reached, all transcripts were reread by the last author (AW) to check if any lived experiences had been missed. Second, to ensure transferability of the findings, the demographics of the participants and the context in which the study was conducted were described with as much detail as possible without jeopardizing anyone’s integrity. Third, to ensure dependability, the third author (MA) conducted the first interview (a pilot interview), and to calibrate subsequent interviews, all 4 authors read it when transcribed and listened jointly to the audio file, after which the interviewing began, following the same structure and interview guide. A majority of the participants chose to be interviewed by telephone instead of in a Zoom video meeting. According to Ward et al,^26^ telephone interviews allow the participants to concentrate on each other’s voice instead of the face, without feeling judged or inhibited. Thus, telephone interviews can be regarded as a valuable option of choice when conducting qualitative research. To ensure confirmability, the first and last author (CG and AW) continuously wrote field notes to raise awareness of preconceived opinions and perceptions that could subjectively influence the analysis process.

## Conclusions

Our findings suggest that the experience of lifelong learning in diabetes self-care in everyday life means learning how to become and remain a lifelong learner in the trajectory of diabetes. Thus, continuous learning of self-care in different situations throughout life helps those diagnosed with a lifelong illness such as diabetes to construct and reconstruct their experiences into a meaningful life. This implies that health care professionals may want not only to proceed from existing guidelines that focus on teaching the patient facts about diabetes but also to support the person’s lifelong learning about diabetes to promote individual diabetes self-care and well-being.
